# Placebo effects in alternative medical treatments for anxiety: false hope or healing potential?

**DOI:** 10.1017/S1092852925100515

**Published:** 2025-08-26

**Authors:** Álex Escolà-Gascón, Neil Dagnall, Kenneth Drinkwater, Abdrew Denovan, Julián Benito-León

**Affiliations:** 1 Department of Quantitative Methods and Statistics, Comillas Pontifical University, established by the Holy See, Vatican City State; 2 Faculty of Health, Psychology and Social Care, Manchester Metropolitan University, Manchester, UK; 3 School of Psychology, Liverpool John Moores University, Liverpool, UK; 4 Department of Neurology, University Hospital, 12 de Octubre, Madrid, Spain; 5 Instituto de Investigación Sanitaria Hospital 12 de Octubre (Imas12), Madrid, Spain; 6 Centro de Investigación Biomédica en Red sobre Enfermedades Neurodegenerativas (CIBERNED), Madrid, Spain; 7 Department of Medicine, Faculty of Medicine, Complutense University, Madrid, Spain

**Keywords:** Causal illusions, placebo effects, anxiety symptom, alternative therapies, paranormal beliefs

## Abstract

**Objective:**

To investigate whether anxiety reductions attributed to healing crystals reflect placebo responses driven by conditioning and belief-related biases rather than specific therapeutic effects.

**Methods:**

In a randomized, controlled study, 138 adults were classified as believers or nonbelievers in crystal efficacy and assigned to rose quartz (experimental) or a visually matched placebo. Participants followed a standardized 14-day protocol. Anxiety was assessed pre- and post-intervention with the Beck Anxiety Inventory and the Spanish Kuwait University Anxiety Scale. Multilevel *analyses of variance* (ANOVA) and Bayesian models were used to evaluate main effects, interactions, and evidence for treatment specificity.

**Results:**

Anxiety reductions occurred only among believers, regardless of crystal assignment. No differences were detected between groups in primary outcomes, and improvements did not exceed the magnitudes typically associated with placebo responses. Bayesian estimates favored the null hypothesis for specific treatment effects. Preexisting belief strongly predicted perceived efficacy and symptom change, consistent with causal illusions plausibly shaped by conditioning mechanisms. Nonbelievers showed no reliable improvement.

**Conclusion:**

Healing crystals did not demonstrate anxiolytic effects beyond those of the placebo. Symptom change was mediated by expectancy and conditioning, particularly in individuals inclined toward intuitive or magical thinking. Although nonspecific, context-dependent factors—such as elements of the therapeutic alliance—may amplify placebo responsiveness in clinical settings, these findings do not support attributing inherent therapeutic value to crystals. Future work should delineate how expectations, clinician-patient rapport, and related variables interact to shape placebo response and how such mechanisms might be ethically leveraged to enhance evidence-based care without promoting pseudoscientific practices.

## Introduction

Classical conditioning is a physiological and psychological model that explains the basic processes of acquisition and behavior modification.^1^ It is based on the association between *unconditioned stimuli* (from now on US), *neutral stimuli* (from now on NS), and *conditioned stimuli* (from now on CS). Each stimulus and its possible combinations can generate two types of responses: *unconditioned responses* (hereafter UR) and *conditioned responses* (hereafter CR).^2^ According to the theory of classical conditioning, learned human behavior originates from relationships between antecedent stimuli and CR.^3^ Currently, classical conditioning is applied in conjunction with *instrumental conditioning*, and both represent the two most effective behavioral models for explaining the antecedents and consequences of human behavior.^4^ ^,^^5^

Initially, classical conditioning was a learning model aimed at stimulating and inhibiting basic behaviors (eg, vegetative).^6^ ^,^^7^ However, later studies have suggested that the limits of the application of classical conditioning in the understanding of human behavior are dynamic.^8^ ^–^^10^ For example, several medical studies have adjusted various models based on CR observed in the immune and endocrine systems.^11^ ^,^^12^ Other research has presented classical conditioning models that explained the psychological mechanisms of the placebo effect.^13^ ^,^^14^ Furthermore, this research bases the search for biological markers of the placebo effect on the principles and applications of classical conditioning.^15^ The versatility of traditional conditioning can also be observed in other studies, which explain and justify the mental programming and deprogramming procedures of sect victims.^16^ In the psychiatric field, classical conditioning has also been used to explain the effectiveness of behavioral therapies in treating post-traumatic stress.^17^ It could therefore be said that the scientific justification of the effectiveness of behavioral treatments would not have been possible without the basic principles of classical conditioning.^18^

One issue that has generated controversy in the medical field is the scientific basis and evidence regarding the efficacy of alternative therapies.^19^ ^,^^20^ A large sector of the scientific community considers alternative therapies as pseudoscientific treatments because they do not meet the guarantees and requirements of the scientific method.^21^ ^,^^22^ In contrast, other health professionals accept that some alternative therapies may be effective and even provide statistical evidence of their clinical efficacy.^23^ ^,^^24^ This is the case for alternative therapies focused on the field of mental health and psychological well-being.^25^ The scientific community that accepts them also considers them to be complementary treatments to traditional medicine, and for this reason, they are called *Complementary and Alternative Medicine* (hereafter CAM).^26^ ^,^^27^ However, no official medical consensus or standard categories exist to classify these therapies as “alternatives”.^28^ Moreover, each country or region has its own legislation regulating the practice of alternative therapies, although the legislation is not always based on published scientific evidence.^29^

As reported previously, the scientific method can be applied at multiple levels, but in all of them, different empirical indicators of the phenomena recorded are analyzed either directly or indirectly. One of these levels consists of identifying and verifying the causal mechanisms that produce certain changes in human behavior.^30^ This is one of the problems of alternative therapies: Significant results can be found in favor of the effectiveness of some therapies (eg, “reiki” or “homeopathy”),^31^ ^–^^33^ but the causal mechanisms that explain the effectiveness are unknown.^34^ In other cases, scientific replications were not satisfactory because they did not outperform the placebo effect.^35^

Despite the massive media campaigns launched in the European Union against pseudosciences, many people believe in their supposed goodness, and many professionals defend their medical usefulness.^36^ ^,^^37^ In addition, the significant results obtained in some investigations, the causes of which remain unknown, have yet to be explained.^34^ ^,^^35^ If there are publications with statistical data in favor of the supposed efficacy of some alternative therapies, rational denial that discredits the scientific validity of these therapies will be insufficient.^38^ The use of the scientific method is necessary to offer “rational alternatives to pseudosciences.”

### Research objectives

This study analyzed and established the therapeutic efficacy of *healing crystals* for the inhibition of anxiety and stress symptoms. Note that some pseudoscientific therapies use crystals as an “energetic treatment” to produce changes in some psychiatric symptoms (eg, reflexology). As a specific objective, it is important to highlight that this research is grounded in the classical conditioning theory as an empirical and psychological model to explain why healing crystals can be effective. As a complement, the changes in the anxiety or stress symptoms are also compared with the placebo effect observed.

### Hypothesis concerning the classical conditioning applied model

The hypotheses of the conditioning models used in this research are derived from the cognitive *theory of dual process.* ^39^ According to this theory, believers in the power of crystals use intuitive and magical information processing, whereas nonbelievers employ a cognitive-rational processing style. This intuitive cognitive style facilitates the association between magical beliefs and the use of healing crystals that produces the causal attribution that “the crystal works.” This attribution is known as *causal illusion.* ^40^

**Figure 1.**
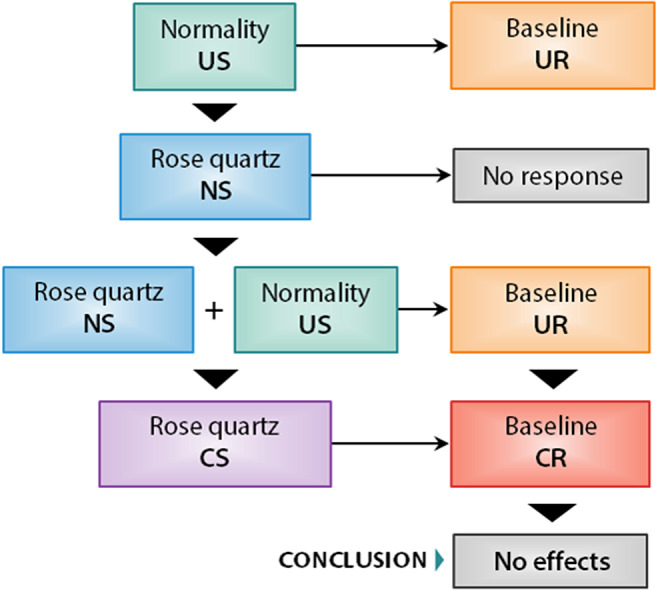
Basic hypothetical model of classical conditioning applied to nonbelievers using rational information processing. In green, the unconditioned stimuli are specified; in blue, the neutral stimuli; in lilac, the conditioned stimuli; in orange, the unconditioned responses; and in red, the conditioned responses. Note that “normality” could be also classified as a set of *conditioned stimuli* (CS). To avoid confusions, in this research, the category “normality” should be understood as the daily events that generate a certain baseline by default. Therefore, this category is considered an unconditioned stimulus because the subject’s response happens automatically.

**Figure 2.**
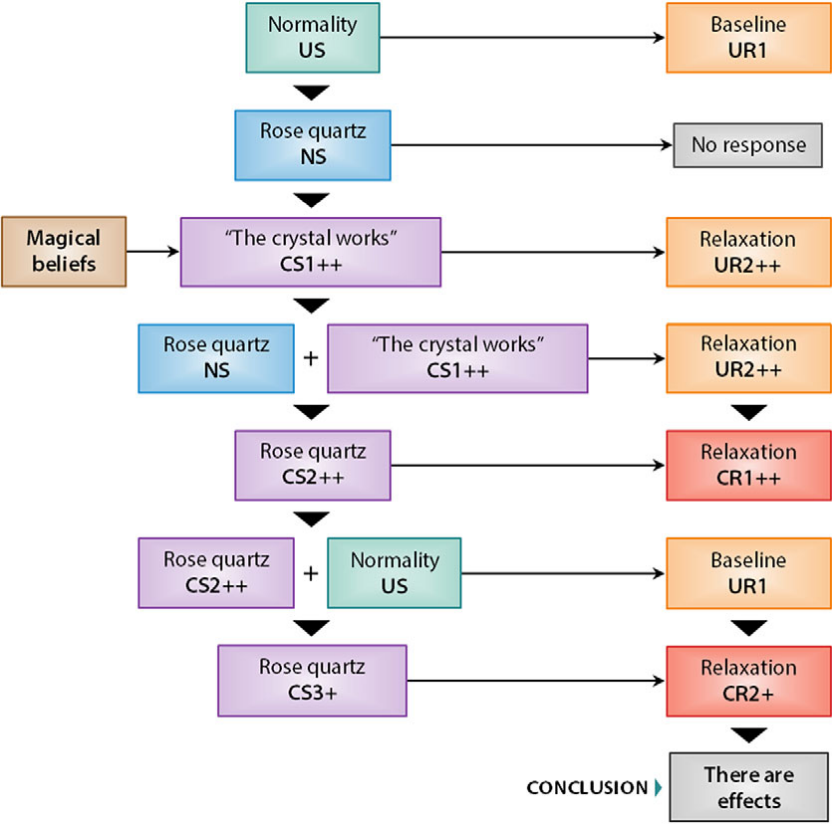
Basic hypothetical model of classical conditioning applied to believers in mineral magic and using irrational information processing. In green, the unconditioned stimuli are specified; in blue, the neutral stimuli; in lilac, the conditioned stimuli; in orange, the unconditioned responses; in red, the conditioned responses; and in brown, the magical beliefs preceding the CS1 stimulus. Note that “normality” could be also classified as a set of *conditioned stimuli* (CS). To avoid confusions, in this research, the category “normality” should be understood as the daily events that generate a certain baseline by default. Therefore, this category is considered an unconditioned stimulus because the subject’s response happens automatically.

In this context, magical beliefs (the irrational processing of information, according to the dual process theory) act as a conditioner that generates the conditioned stimulus “the crystal works.” This stimulus is an internal cognition that generates a sense of security or control. This perception of control induces somatic relaxation and lowers the anxiety/stress levels of the participants. The hypothetical models are presented in Figures 1 and 2.

The figures above describe the mechanisms by which the use of healing crystals could reduce anxiety levels. On the one hand, Figure 1 reflects the associations for participants using rational information processing. Given that no conditioned stimulus intervenes to modify the individual’s base response, the individual does not attribute healing crystals. On the other hand, Figure 2 shows the associations for participants who use irrational information processing. In this case, the conditioner is the magical beliefs that justify the final causal illusion that healing crystals are effective.

Therefore, the formal hypotheses were:

(1) Believing participants who use irrational cognitive processing will attribute effectiveness to healing crystals, and their anxiety-stress levels will decrease through somatic action after the use of healing crystals. (2) Nonbelievers who use rational cognitive processing will not attribute efficacy to the healing crystals, and their anxiety-stress levels will not change significantly after the use of the healing crystals. (3) The pre- and post-differences between the experimental groups will not exceed the differences observed between the pre- and post-groups who received a placebo.

## Methods

### Participants

A total of 138 participants from the general nonclinical population collaborated; 54% were men and 46% were women. All were adults (mean age = 34.90; standard deviation = 7.29). Seventy participants were believers in pseudosciences and specifically in the healing power of crystals, 68 were nonbelievers. In the procedures subsection, we explain how these participants were classified. All participants signed an informed consent form by which they authorized their collaboration in the experiment and the analysis of the results. Similarly, the participants in the sample self-reported that they did not suffer or had not suffered from any mental disorder officially diagnosed by a medical doctor. All had an active working life and stated that they did not have severe financial difficulties.

### Procedures

This study used an experimental design that applied the recommended multilevel methodology for this type of study.^41^ Both the sampling and the development of the phases of the experiment were carried out in accordance with the hypotheses and models of classical conditioning set out in the introduction.

#### Development of the sampling

**Figure 3.**
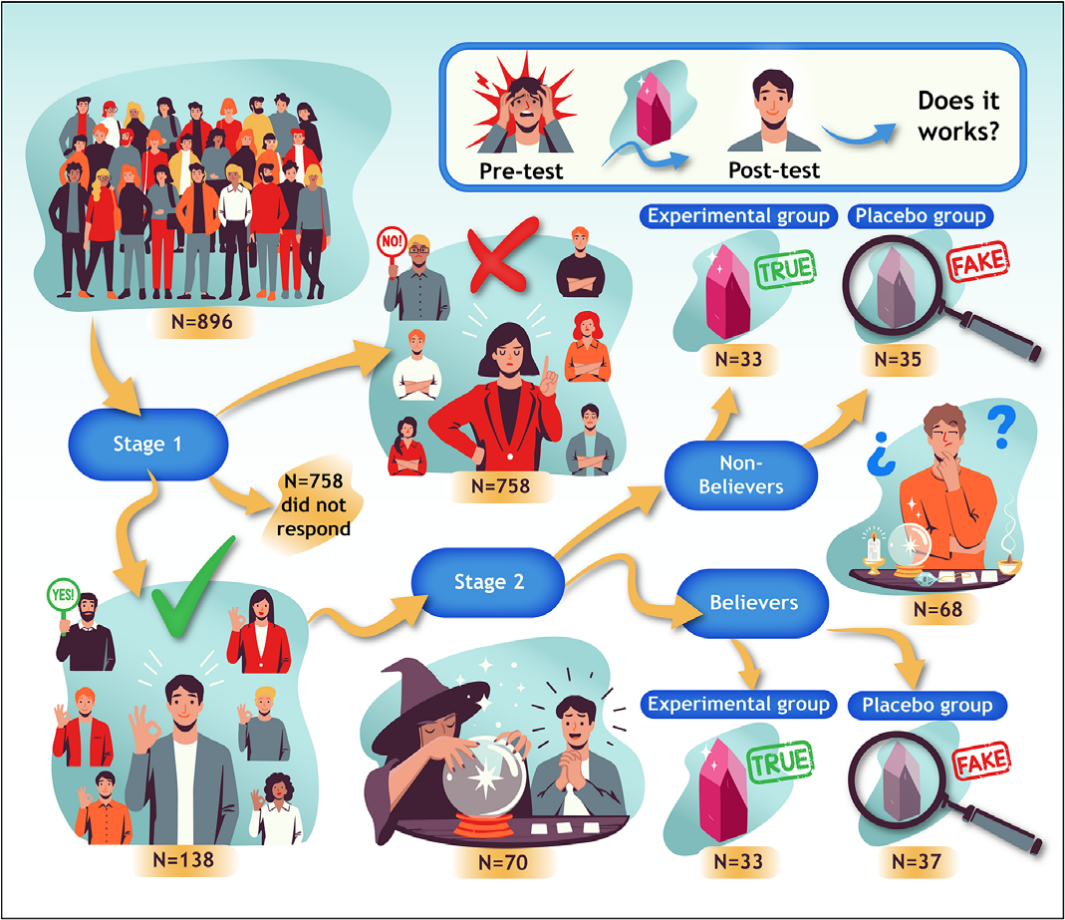
Participant selection process and group distribution. The notation “(N = 68)” in the central right section of the figure refers to the 68 nonbeliever participants who were randomly assigned to the experimental or control condition.

In a previous study conducted at the MAGIC International alternative therapies fair, participants who volunteered provided their email address. Using the contact list, a message was written notifying a total of 896 participants of the possibility of collaborating in an experimental study related to the use of healing crystals. Two weeks after the e-mail was sent, 153 participants responded and agreed to collaborate. The remaining 743 either responded negatively to the proposal or did not respond. Figure 3 schematically summarizes the successive steps taken during the sampling.

A further e-mail was then sent to the 153 participants specifying the conditions of the research and the informed consent. The conditions were based on the **exclusion criteria**, which were: (1) having no history of a formally diagnosed mental disorder; (2) being free from any serious chronic disease (those medically related to anxiety-stress were considered, including neurodegenerative diseases, cardiac conditions, neuropathies, substance dependencies, and chronic-pain-related illnesses); (3) not being affected by any terminal illness; (4) having no history of COVID-19 infection (given its strong association with stress and anxiety due to its socio-economic impact); and (5) explicitly reporting a situation of personal risk or a borderline condition as a result of the COVID-19 crisis (eg, job layoffs, divorces, evictions, job changes, or any situation that could be a source of stress for the participant).

Of the 153 participants, 15 contacted the researcher again, notifying them that they met one of the exclusion criteria and were therefore excluded from the research. Thus, 70 participants remained who were believers, and another 68 were nonbelievers. The believers and nonbelievers were randomly distributed into two groups: the *control group* or CG (who would receive a placebo) and the *experimental group* or EG (who would receive a crystal healing). We must emphasize that the distinction between these two groups allowed us to identify those whose responses were conditioned by their beliefs (the believers) and those who did not hold a belief in the effectiveness of crystal healing. Therefore, it should be understood that rationally, the stimulus diagram in Figure 2 is the one that applies to the believers group.

The differentiation between believing and nonbelieving participants was made based on market research conducted for the MAGIC International trade fair. In this study, each visitor to MAGIC was asked to voluntarily answer questionnaires related to pseudoscientific beliefs. The scores on the questionnaire measuring beliefs in alternative therapies (specifically, the median was used as a statistical criterion) were used to discriminate believers versus nonbelievers. Those who scored above the median were classified as believers, and those who scored below were classified as nonbelievers. In this way, it was possible to identify in which participants there was a causal illusion that alternative therapies work and in which there was not. This was an essential procedure that ensured that when classical conditioning theory (believers) was present, and when nonbelievers were not present in the volunteers in this research.

#### Phases of the experiment

Participants were assembled in separate sessions based on their assigned group classification, and the experimental instructions were provided accordingly. The phases were the same for each group: (1) explanation of the instructions containing the following guidelines:

*We will give you some envelopes containing natural crystals. According to certain ancestral beliefs, crystals can have healing properties if they are used in a certain way. We ask that during the next two weeks (14 days and 13 nights), you comply with the instructions we provide below. At night: Immerse the rose crystal in water with salt and keep it submerged all night long. In the morning: Take it out of the salt water, dry it, and keep it in your wallet. If it does not fit in your purse, you can keep it anywhere or in anything that goes with you at all times. The important thing is that the mineral is close to you throughout the day. When you arrive home the next night, repeat the washing process. General considerations: You can touch the rose crystal, but do not let others see or touch it. You can keep the crystal inside the envelope or wrapped in a handkerchief if you want to be discreet. Avoid being influenced by others; do not tell anyone why you use the crystal or what your intentions are regarding its use (you can notify those around you that you are participating in scientific research related to the use of crystals, but try not to tell anyone else). Follow these guidelines for the two weeks. If you forget to do the morning or evening instructions one day, you must notify the researcher of this project. Do not interrupt this activity at any time. If you wish to stop the experiment for private reasons, you must notify the researcher immediately, and you will be removed from the research, but do not do so on your own. Finally, please inform the coordinator of this research if you had an unexpected boundary situation during the experimental period (eg, death of a family member). In these cases, the participant will be immediately removed from the research.*

***Remember that this activity does have negative repercussions for you.** At the end of the experimental period, we will give you the rose crystal as a thank you for your collaboration in this research. Do not discard these instructions since you may need to read them in the next few days.*

**Figure 4.**
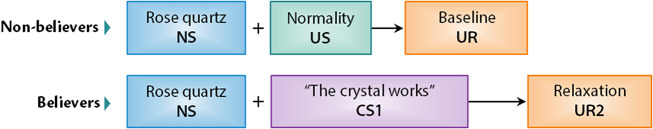
Differential associations between believers and nonbelievers. Note that “normality” could be also classified as a set of *conditioned stimuli* (CS). To avoid confusions, in this research, the category “normality” should be understood as the daily events that generate a certain baseline by default. Therefore, this category is considered an unconditioned stimulus because the subject’s response happens automatically.

Phase continuation: (2) signing of the informed consent; (3) application of the anxiety-stress tests, and the following question was asked: ─ On a scale from 1 to 10 (with 1 being “not at all” and 10 being “to the maximum”), how much do you think rose quartz will contribute to your personal well-being? (All these questionnaires represented the pre-test application); (4) handing out the materials to the participants. The EG was given a small portion of polished rose quartz, while the CG was given a small rose decorative stone that simulated the original rose quartz. These stones were purchased from the *Garden Center, Inc.* and were solid glass pebbles for vases, flowers, or centerpieces. Neither the mineral nor the glass pebbles were harmful or toxic to the touch. (5) A new optional meeting was arranged with the participants at the end of the two weeks in case they wanted to share their experience with each other. It should be noted that after the 14 days, the participants received the questionnaires and digitized surveys by e-mail for them to answer again (post-test). The following question was also asked again: On a scale of 1 to 10 (1 being “not at all” and 10 being “at most”), how much do you think rose quartz has contributed to your personal well-being? This question and the same version, but applied in the pre-test, were intended to quantify the associations in Figure 4.

These associations correspond to the models of classical conditioning hypothesized in Figures 1 and 2. Believers had to select pre- and post-test values greater than 5 to ensure that the neutral stimulus “rose quartz” was associated with the conviction of “the crystal works” (see Figure 2). On the other hand, following this logic, nonbelievers had to mark values below 5.

Since at the end of the experiment the control participants were notified that they had received a placebo, they were also given the possibility of receiving a real rose quartz as a gift in compensation. In this way, they would be on an equal footing with the participants in the EG at the end of the research.

### Instruments

#### Beck Anxiety Inventory

The *Beck Anxiety Inventory* (BAI) was developed for the assessment or screening of anxiety-related symptoms, both clinical and subclinical.^42^ This scale has 21 items, grouped into two dimensions: somatic anxiety and affective anxiety. The participant indicated the frequency with which he or she perceived each of the described symptoms. In this study, the Spanish version of the BAI was used, with responses scored on a Likert scale from 0 (“not at all”) to 3 (“severely; I could barely stand it”).^43^ This questionnaire is widely used in psychiatric evaluations and presents guarantees of its validity and reliability.^42^ The reliability indices for this sample based on internal consistency were satisfactory for each dimension (alpha>0.8 and omega coefficient > 0.8).

#### Spanish Kuwait University Anxiety Scale

The *Spanish Kuwait University Anxiety Scale* (S-KUAS) is a psychometric inventory specially designed to assess anxiety symptoms in the general nonclinical population.^44^ In this scale, the participant also indicated the frequency with which he or she perceived the symptoms specified in the items. The S-KUAS consists of 20 items distributed in three dimensions: *Subjective Anxiety* (7 items), *Cognitive Anxiety* (9 items), and *Somatic Anxiety* (4 items). All responses are also coded using a Likert scale ranging from 1 (“rarely”) to 4 (“always”). The Spanish adaptation was used in this study, which presents sufficient evidence of the reliability and validity of this test.^45^ In fact, its reliability indices are largely satisfactory (>0.8), and it has a consistent internal structure at the factorial level. In our sample, the reliability of the dimensions and scores of this scale was also acceptable (alpha>0.7 and omega coefficient > 0.7).

### Data analysis

Data were processed and analyzed with the *Jeffreys’s Amazing Statistics Program* (JASP) and Jamovi software.^46^ Both classical frequency analysis models and a Bayesian approach based on *Bayes Factor* (hereinafter BF) were used. Specifically, a multilevel 3-factor *analysis of variance* (ANOVA) model was applied.

**Figure 5.**
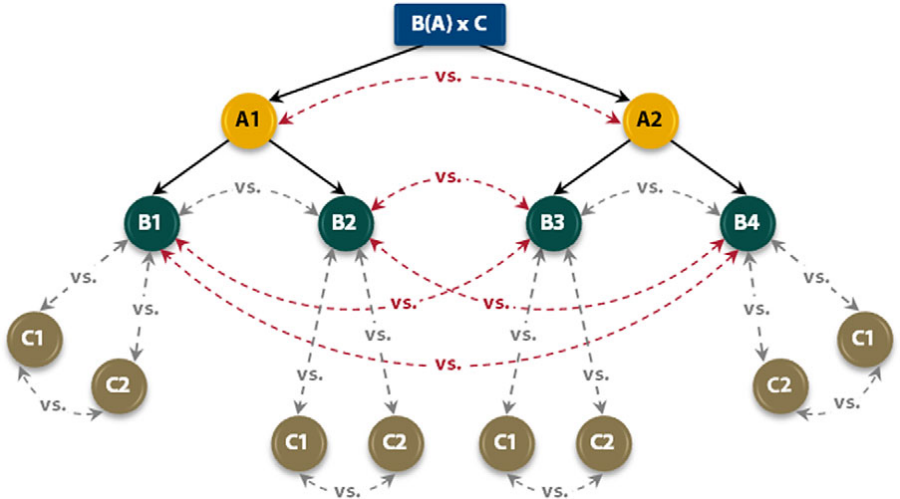
Network plot with multilevel comparisons utilized in this research (see red). Note that multilevel comparisons include C1 and C2 groups, but there is not enough space to draw the red arrows. The group comparisons at the same level are in gray. Important warning: Consider “B” as the experimental independent variable (where B1 = control group and B2 = experimental group); “A” as the belief systems variable (where A1 = believers and A2 = nonbelievers); “C” as the longitudinal measurements (where C1 = pre-test and C2 = post-test). The bracket means that variable B is nested in variable A. Clarification: variable “B” has different nested groups in variable “A.” Therefore, the interaction A 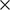 B cannot be carried out in this multilevel design, since B1 is not nested in A2, for instance.

The first was the *nesting factor* (called “A”) and distinguished two levels: the participants who believed in the magic energy of crystals (level A1) and the nonbelievers (level A2). The second factor (called “B”) was *completely randomized* into four levels: B1 = control group of believers; B2 = experimental group of believers; B3 = control group of nonbelievers; and B4 = experimental group of nonbelievers. Finally, the third level distinguished the two *longitudinal measures* (called “C”), which were C1 = pre-test and C2 = post-test. Using the algebraic expressions, this design can be represented as follows: B(A) 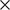 C. The nesting variable is specified in parentheses. To facilitate the understanding of this multilevel model, Figure 5 illustrates the comparisons and effects that were analyzed in this research.

As for the Bayesian estimation, two types of probabilities were estimated to obtain the BFs: (1) the probability that these data fit the distribution relative to the null hypothesis (*H*_0_) (*H*_0_ = the results are not significant), represented as 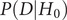 , and (2) the probability that these data fit the distribution relative to the alternative hypothesis (*H*_1_), represented as 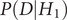 . Unlike *likelihood ratios*, Bayesian analyses estimate the above probabilities using integration procedures. In this case, the following equation was applied:
(1)

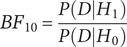



The BFs can be transformed to obtain the probabilities a posteriori. Specifically, in this research, we wanted to obtain the probability that the alternative hypothesis fits the sample, which is represented as 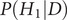 . When the a priori probabilities are adjusted to 50%, the following formula can be used:
(2)

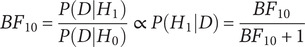



Considering the comparisons specified in Figure 5, it is important to add that the interactions A 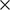 C and B 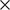 C were the only interactions that could be calculated, since the levels of the variable “B” were different from each other. This may generate confusion, since the CGs and EGs have the same labels in both A1 and A2. However, they are not the same because the nesting variable “A” is not a random-effects variable, so the characteristics of B1 and B2 cannot be the same as the characteristics of B3 and B4. If the nesting variable had been random-effects, then levels B1 and B2 would be equivalent to groups B3 and B4. Only in the latter scenario would it make sense to analyze the A 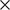 B interaction, but this is not the case in this research.

## Results

### Descriptive analyses and multilevel ANOVA models

Descriptive statistics are provided in Tables 1 and 2. Note that the 3-factor ANOVA model was based on the contrast of marginal means (see Table 1). Simple effects, simple interaction effects, and multilevel interaction effects were based on the comparison of observed means for each variable and group.^47^

**Table 1.** Descriptive Statistics for Only Variables (marginal means)

	Belief systems		Experimental treatments		Measurements (*N* = 138)	
DV	*Believers* (*N* = 70)	*Nonbelievers* (*N* = 68)	CG	EG	Pre	Post
	*M*	*M*	*M*	*M*	*M*	*M*
*BA*	4.836	4.836	4.935	4.717	5.127	4.526
	(0.354)		(0.354)		(0.253)	
*BS*	9.680	11.197	10.133	10.744	10.774	10.103
	(0.704)		(0.709)		(0.501)	
*KS*	7.133	7.172	7.196	7.108	7.397	6.908
	(0.195)		(0.195)		(0.141)	
*KC*	14.119	14.344	14.621	13.843	14.419	14.045
	(0.395)		(0.393)		(0.282)	
*KSU*	13.325	12.530	12.771	13.084	13.085	12.770
	(0.434)		(0.436)		(0.308)	

*Note:* Standard deviations are in brackets.

Abbreviations: DV = dependent variables; M = means; SD = standard deviation; CG = control group; EG = experimental group; BA = BAI affective anxiety; BS = BAI somatic anxiety; KS = S-KUAS somatic anxiety; KC = S-KUAS cognitive anxiety; KSU = S-KUAS subjective anxiety.

**Table 2.** Descriptive Statistics per Variables and Groups

LM	Dependent variables	Believers				Nonbelievers			
		Control group (*N* = 33)		Experimental group (*N* = 35)		Control group (*N* = 33)		Experimental group (*N* = 37)	
		*M*	*SD*	*M*	*SD*	*M*	*SD*	*M*	*SD*
*Pre*	*BA*	5.33	3.379	5.46	2.694	4.55	3.260	5.14	3.237
	*BS*	11.45	5.837	11.73	6.270	10.58	6.408	9.31	6.115
	*KS*	7.39	1.560	7.70	1.614	7.18	1.629	7.29	1.824
	*KC*	14.55	3.001	14.73	2.845	13.39	3.455	14.94	3.880
	*KSU*	12.73	3.867	12.86	3.417	13.64	3.991	13.14	3.631
*Post*	*BA*	4.48	2.917	4.08	2.005	4.48	3.094	5.06	3.199
	*BS*	10.94	5.338	10.62	5.459	10.06	6.108	8.80	5.671
	*KS*	6.79	1.556	6.78	1.669	7.06	1.749	7	1.663
	*KC*	14.15	3.012	13.95	2.624	13.21	3.507	14.83	3.507
	*KSU*	12.48	3.641	12.08	3.130	13.52	3.874	13.06	3.589

Abbreviations: M = means; SD = standard deviation; BA = BAI affective anxiety; BS = BAI somatic anxiety; KS = S-KUAS somatic anxiety; KC = S-KUAS cognitive anxiety; KSU = S-KUAS subjective anxiety.

As can be seen in Table 2, the means for the group of believers (both experimental and control) tended to be higher than the means for the group of nonbelievers. However, these increases should be analyzed both from the marginal means and from the observed means related to the simple effects. To better understand the analysis of simple effects, Table 3 is provided.

**Table 3.** Example of a *Multilevel* Contingency Table with the Location of Each cell. In each cell, there will be the mean corresponding to each dependent variable (see Table 1)

Nesting variable	Nested groups	**C1**- *Pre-test*	**C2**- *Post test*	Marginal means
**A1**- *Believers*	**B1**- *Control*	B1(A1) 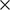 C1	B1(A1) 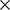 C2	B1(A1) 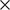 C+
	**B2**- *Experimental*	B2(A1) 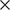 C1	B2(A1) 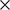 C2	B2(A1) 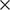 C+
**A2**- *Nonbelievers*	**B3**- *Control*	B3(A2) 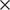 C1	B3(A2) 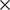 C2	B3(A2) 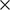 C+
	**B4**- *Experimental*	B4(A2) 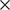 C1	B4(A2) 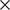 C2	B4(A2) 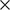 C+
	Marginal means	B+(A+) 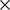 C1	B+(A+) 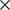 C2	B+(A+) 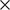 C+

Important warning: all matrix notations in each cell are based on the algebraic expression [B(A) 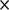 C], where: B = is the experimental independent variable; A = is the belief systems variable, and C = are longitudinal measurements. The bracket means that variable B is nested in variable A (see Figure 5 for more information).

This table summarizes the relationship between the three variables used. Both the CG and the EG are nested categories within the believing and nonbelieving groups. The algebraic expressions are equivalent to the means in Table 2. For example, the expression 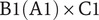 represents the mean that summarizes the scores of the participants belonging to the CG in the nested category of believers and in the pre-test measure. This logic should be applied to the rest of the boxes. It should be considered that there will be as many means as cells and dependent variables. The ANOVA contrast and the Bayesian estimation of the main effects of the variables “A” (beliefs), “B” (treatment variable), and “C” (pre- and post-measures) as well as their respective A 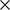 C and B 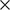 C interactions, are presented in Table 4.

**Table 4.** Analysis of variance, Main Effects of Variables, and Bayesian Approach

DV	IV	*F*	*p*	*BF_10_* (% estimated error)	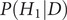	R^2^
*BAI- affective*	A	0.004	0.949	0.472 (error % = 4.052)	0.472	N.S.
	B	0.197	0.658	0.327 (error % = 4.707)	0.246	N.S.
	C	54.317	<0.001	8.043e+6 (error % = 1.022)	∼1	0.926
	A 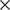 C	41.949	<0.001	5.675e+6 (error % = 7.543)	∼1	0.937
	B 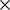 C	16.177	<0.001	2.2e+6 (error % = 9.089)	∼1	0.916
*BAI- somatic*	A	2.321	0.130	0.441 (error % = 4.675)	0.306	N.S.
	B	1.028	0.382	0.332 (error % = 3.204)	0.249	N.S.
	C	34.279	<0.001	297670.421 (error % = 0.853)	∼1	0.966
	A 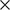 C	1.871	0.174	0.374 (error % = 12.276)	0.272	N.S.
	B 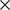 C	1.768	0.156	0.422 (error % = 21.964)	0.297	N.S.
*S-KUAS- somatic*	A	0.020	0.887	0.398 (error % = 3.596)	0.285	N.S.
	B	0.059	0.981	0.227 (error % = 4.369)	0.185	N.S.
	C	72.901	<0.001	9.656e+9 (error % = 1.307)	∼1	0.896
	A 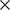 C	24.412	<0.001	6221.149 (error % = 6.681)	∼1	0.907
	B 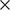 C	9.943	<0.001	2991.182 (error % = 8.285)	∼1	0.885
*S-KUAS- cognitive*	A	0.162	0.688	0.613 (error % = 4.681)	0.380	N.S.
	B	1.391	0.248	0.384 (error % = 6.190)	0.277	N.S.
	C	25.837	<0.001	6996.531 (error % = 1.275)	∼1	0.956
	A 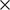 C	9.498	0.002	13.940 (error % = 8.500)	0.933	0.954
	B 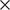 C	4.442	0.005	7.584 (error % = 15.658)	0.884	0.943
*S-KUAS- subjective*	A	1.681	0.197	0.394 (error % = 4.851)	0.283	N.S.
	B	0.659	0.579	0.547 (error % = 5.488)	0.354	N.S.
	C	27.625	<0.001	11555.168 (error % = 1.507)	∼1	0.972
	A 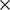 C	12.549	<0.001	36.880 (error % = 16.036)	∼1	0.968
	B 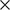 C	8.123	<0.001	387.166 (error % = 8.584)	0.997	0.963

Abbreviations: B = is the experimental independent variable; A = is the belief systems variable and C = are longitudinal measurements; DV = dependent variables; IV = independent variables; F = Fisher’s tests; BF_10_ = Bayes factors in favor of the alternative hypothesis; R^2^ = explained variance corrected according BFs.

Clarification: Variable “B” has different groups nested in variable “A.” Therefore, the interaction A × B cannot be carried out in this multilevel design, since B1 is not nested in A2, for instance.

Significant results were obtained only for variable “C” and the respective interactions. The BFs also supported these results, as they were higher than 10 for most variables.^48^ The main effects of variables “A” and “B” showed no significant differences.

Since the “C” variable and the interactions had effects on the dependent variables, we analyzed the simple effects and the multilevel interaction effects of the B(A) nesting in Tables 5 through 10. It is important to note that in this contrast, the post-hoc comparisons could not be applied since there were only 2 groups for each independent variable. The algebraic expressions in Table 3 specify which comparisons were made between the mean boxes for each measured dependent variable (see BAI and S-KUAS anxiety scales).

**Table 5.** Simple main and interaction effects Analysis for the Pretests

DV	Means comparison	t-test**	*p* values (Tukey)	*p* values (Bonferroni)	d
*BA*	B1(A1) 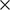 C1 versus B2(A1) 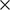 C1 B1(A1) 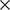 C1 versus B3(A2) 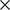 C1* B1(A1) 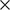 C1 versus B4(A2) 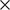 C1* B2(A1) 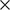 C1 versus B3(A2) 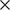 C1* B2(A1) 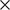 C1 versus B4(A2) 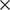 C1* B3(A2) 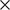 C1 versus B4(A2) 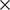 C1	−0.168 1.019 0.250 1.215 0.427 −0.784	0.998 0.739 0.995 0.619 0.974 0.862	∼1 ∼1 ∼1 ∼1 ∼1 ∼1	−0.042 0.237 0.058 0.307 0.107 −0.184
*BS*	B1(A1) 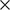 C1 versus B2(A1) 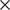 C1 B1(A1) 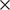 C1 versus B3(A2) 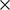 C1* B1(A1) 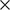 C1 versus B4(A2) 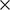 C1* B2(A1) 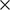 C1 versus B3(A2) 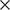 C1* B2(A1) 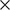 C1 versus B4(A2) 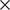 C1* B3(A2) 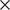 C1 versus B4(A2) 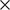 C1	−0.186 0.579 1.431 0.782 1.662 0.843	0.998 0.938 0.482 0.863 0.348 0.834	∼1 ∼1 0.928 ∼1 0.593 ∼1	−0.045 0.143 0.358 0.182 0.390 0.202
*KS*	B1(A1) 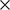 C1 versus B2(A1) 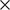 C1 B1(A1) 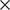 C1 versus B3(A2) 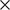 C1* B1(A1) 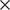 C1 versus B4(A2) 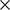 C1* B2(A1) 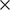 C1 versus B3(A2) 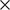 C1* B2(A1) 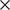 C1 versus B4(A2) 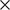 C1* B3(A2) 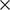 C1 versus B4(A2) 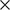 C1	−0.776 0.519 0.269 1.310 1.065 −0.258	0.865 0.954 0.993 0.558 0.712 0.994	∼1 ∼1 ∼1 ∼1 ∼1 ∼1	−0.194 0.133 0.064 0.321 0.243 −0.060
*KC*	B1(A1) 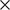 C1 versus B2(A1) 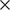 C1 B1(A1) 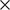 C1 versus B3(A2) 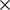 C1* B1(A1) 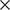 C1 versus B4(A2) 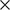 C1* B2(A1) 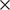 C1 versus B3(A2) 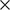 C1* B2(A1) 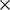 C1 versus B4(A2) 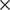 C1* B3(A2) 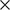 C1 versus B4(A2) 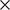 C1	−0.232 1.411 −0.494 1.683 −0.273 −1.925	0.996 0.495 0.960 0.337 0.993 0.222	∼1 0.964 ∼1 0.569 ∼1 0.338	−0.063 0.356 −0.114 0.425 −0.063 −0.421
*KSU*	B1(A1) 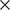 C1 versus B2(A1) 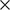 C1 B1(A1) 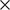 C1 versus B3(A2) 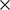 C1* B1(A1) 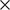 C1 versus B4(A2) 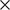 C1* B2(A1) 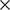 C1 versus B3(A2) 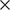 C1* B2(A1) 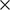 C1 versus B4(A2) 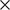 C1* B3(A2) 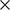 C1 versus B4(A2) 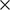 C1	−0.154 −0.992 −0.460 −0.866 −0.317 0.546	0.999 0.754 0.968 0.823 0.989 0.947	∼1 ∼1 ∼1 ∼1 ∼1 ∼1	−0.038 −0.231 −0.111 −0.209 −0.079 0.130

*Note:* *simple interaction multilevel effects; **t-test was corrected for multiple comparisons. Important warning: all means comparisons come from the matrix annotations of Table 3.

Abbreviations: DV = dependent variables; BA = BAI affective anxiety; BS = BAI somatic anxiety; KS = S-KUAS somatic anxiety; KC = S-KUAS cognitive anxiety; KSU = S-KUAS subjective anxiety; d = Cohen’s d corrected using Hedges’ g.

The simple effects of differences between the CG and the EG nested in the categories of believers and nonbelievers were not significant when comparisons were made within the pre- and post-measures separately (see Tables 5 and 6). However, significant results were obtained for the single effects that compared the pre and post scores of the nested control participants in the category “believers.” Significant results were also obtained for comparisons between means of pre and post scores of experimental participants nested in the category “nonbelievers” (see Tables 7, 8, 9, and 10).

**Table 6.** Simple Main and Interaction Effects Analysis for the Post Tests

DV	Means comparison	t-test**	*p* values (Tukey)	*p* values (Bonferroni)	d
*BA*	B1(A1) 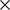 C2 versus B2(A1) 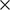 C2 B1(A1) 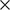 C2 versus B3(A2) 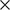 C2* B1(A1) 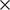 C2 versus B4(A2) 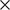 C2* B2(A1) 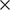 C2 versus B3(A2) 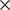 C2* B2(A1) 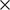 C2 versus B4(A2) 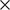 C2* B3(A2) 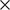 C2 versus B4(A2) 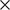 C2	0.596 4.715e − 15 −0.834 −0.596 −1.464 −0.834	0.933 ∼1 0.838 0.933 0.462 0.838	∼1 ∼1 ∼1 ∼1 0.873 ∼1	0.163 - −0.187 −0.157 −0.368 −0.182
*BS*	B1(A1) 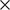 C2 versus B2(A1) 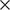 C2 B1(A1) 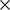 C2 versus B3(A2) 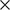 C2* B1(A1) 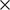 C2 versus B4(A2) 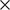 C2* B2(A1) 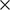 C2 versus B3(A2) 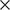 C2* B2(A1) 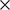 C2 versus B4(A2) 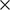 C2* B3(A2) 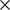 C2 versus B4(A2) 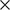 C2	0.235 0.632 1.562 0.415 1.368 0.920	0.995 0.921 0.404 0.976 0.521 0.794	∼1 ∼1 0.724 ∼1 ∼1 ∼1	0.059 0.153 0.388 0.097 0.327 0.214
*KS*	B1(A1) 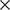 C2 versus B2(A1) 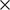 C2 B1(A1) 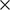 C2 versus B3(A2) 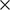 C2* B1(A1) 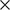 C2 versus B4(A2) 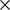 C2* B2(A1) 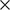 C2 versus B3(A2) 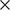 C2* B2(A1) 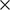 C2 versus B4(A2) 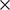 C2* B3(A2) 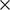 C2 versus B4(A2) 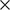 C2	0.010 −0.667 −0.526 −0.696 −0.552 0.150	∼1 0.909 0.953 0.898 0.946 0.999	∼1 ∼1 ∼1 ∼1 ∼1 ∼1	0.003 −0.165 −0.132 −0.162 −0.130 0.036
*KC*	B1(A1) 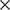 C2 versus B2(A1) 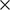 C2 B1(A1) 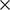 C2 versus B3(A2) 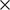 C2* B1(A1) 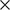 C2 versus B4(A2) 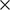 C2* B2(A1) 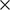 C2 versus B3(A2) 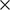 C2* B2(A1) 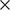 C2 versus B4(A2) 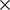 C2* B3(A2) 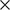 C2 versus B4(A2) 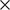 C2	0.264 1.172 −0.857 0.941 −1.150 −2.046	0.994 0.646 0.827 0.783 0.660 0.177	∼1 ∼1 ∼1 ∼1 ∼1 0.256	0.073 0.287 −0.197 0.239 −0.272 −0.442
*KSU*	B1(A1) 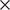 C2 versus B2(A1) 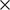 C2 B1(A1) 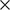 C2 versus B3(A2) 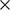 C2* B1(A1) 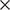 C2 versus B4(A2) 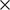 C2* B2(A1) 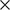 C2 versus B3(A2) 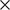 C2* B2(A1) 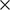 C2 versus B4(A2) 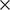 C2* B3(A2) 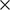 C2 versus B4(A2) 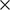 C2	0.474 −1.177 −0.663 −1.684 −1.164 0.531	0.965 0.643 0.911 0.336 0.651 0.951	∼1 ∼1 ∼1 0.567 ∼1 ∼1	0.119 −0.274 −0.158 −0.410 −0.290 0.123

*Note:* **t-test was corrected for multiple comparisons. Important warning: All mean comparisons come from matrix annotations of Table 3.

Abbreviations: DV = dependent variables; BA = BAI affective anxiety; BS = BAI somatic anxiety; KS = S-KUAS somatic anxiety; KC = S-KUAS cognitive anxiety; KSU = S-KUAS subjective anxiety; d = Cohen’s d corrected using Hedges’ g; *simple interaction multilevel effects.

**Table 7.** Simple Main and Interaction Effects Analysis for the Believers

DV	Means comparison	t-test**	*p* values (Tukey)	*p* values (Bonferroni)	d
*BA*	**B1(A1)** 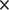 **C1 versus B1(A1)** 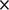 **C2** B1(A1) 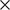 C1 versus B2(A1) 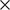 C2 B1(A1) 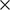 C1 versus B3(A2) 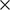 C2* B1(A1) 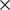 C1 versus B4(A2) 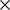 C2* B2(A1) 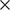 C1 versus B1(A1) 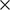 C2 **B2(A1)** 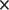 **C1 versus B2(A1)** 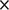 **C2** B2(A1) 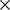 C1 versus B3(A2) 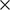 C2* B2(A1) 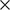 C1 versus B4(A2) 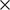 C2*	**−5.160** −1.750 −1.153 −0.381 −1.362 **−8.876** −1.362 −0.571	**<0.001** 0.655 0.943 ∼1 0.873 **<0.001** 0.873 0.999	<**0.001** ∼1 ∼1 ∼1 ∼1 **<0.001** ∼1 ∼1	**−0.439** −0.149 −0.098 −0.032 −0.116 **−0.756** −0.116 −0.049
*BS*	B1(A1) 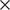 C1 versus B1(A1) 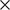 C2 B1(A1) 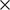 C1 versus B2(A1) 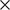 C2 B1(A1) 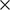 C1 versus B3(A2) 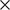 C2* B1(A1) 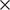 C1 versus B4(A2) 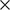 C2* B2(A1) 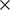 C1 versus B1(A1) 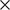 C2 **B2(A1)** 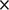 **C1 versus B2(A1)** 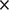 **C2** B2(A1) 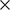 C1 versus B3(A2) 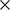 C2* B2(A1) 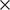 C1 versus B4(A2) 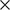 C2*	−2.208 −0.589 −0.958 −1.851 −0.558 −**5.028** −1.179 −2.102	0.354 0.999 0.979 0.587 0.999 **<0.001** 0.937 0.419	0.811 ∼1 ∼1 ∼1 ∼1 **<0.001** ∼1 ∼1	−0.188 −0.050 −0.082 −0.158 −0.048-**0.428** −0.100 −0.179

*Note:* *simple interaction multilevel effects; **t-test was corrected for multiple comparisons. Important warning: All means comparisons come from matrix annotations of Table 3.

Abbreviations: DV = dependent variables; BA = BAI affective anxiety; BS = BAI somatic anxiety; KS = S-KUAS somatic anxiety; KC = S-KUAS cognitive anxiety; KSU = S-KUAS subjective anxiety; d = Cohen’s d corrected using Hedges’ g.

**Table 8.** Simple Main and Interaction Effects Analysis for the Believers (continuation Table 7)

DV	Means comparison	t-test**	*p* values (Tukey)	*p* values (Bonferroni)	d
*KS*	**B1(A1)** 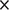 **C1 versus B1(A1)** 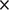 **C2** B1(A1) 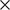 C1 versus B2(A1) 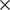 C2 B1(A1) 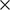 C1 versus B3(A2) 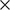 C2* B1(A1) 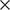 C1 versus B4(A2) 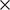 C2* B2(A1) 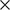 C1 versus B1(A1) 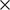 C2 **B2(A1)** 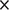 **C1 versus B2(A1)** 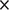 **C2** B2(A1) 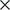 C1 versus B3(A2) 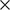 C2* B2(A1) 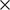 C1 versus B4(A2) 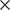 C2*	**−5.234** −1.534 −0.815 −0.977 −2.300 **−8.403** −1.615 −1.794	**<0.001** 0.788 0.992 0.977 0.301 **<0.001** 0.741 0.625	**<0.001** ∼1 ∼1 ∼1 0.640 **<0.001** ∼1 ∼1	**−0.446** −0.131 −0.069 −0.083 −0.196 **−0.715** −0.137 −0.153
*KC*	B1(A1) 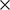 C1 versus B1(A1) 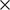 C2 B1(A1) 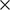 C1 versus B2(A1) 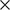 C2 B1(A1) 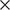 C1 versus B3(A2) 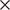 C2* B1(A1) 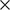 C1 versus B4(A2) 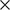 C2* B2(A1) 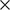 C1 versus B1(A1) 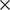 C2 **B2(A1)** 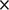 **C1 versus B2(A1)** 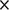 **C2** B2(A1) 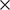 C1 versus B3(A2) 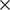 C2* B2(A1) 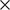 C1 versus B4(A2) 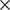 C2*	−2.638 −0.762 −1.648 0.355 −0.735 **−5.558** −1.929 0.128	0.152 0.995 0.720 ∼1 0.996 **<0.001** 0.534 ∼1	0.261 ∼1 ∼1 ∼1 ∼1 **<0.001** ∼1 ∼1	−0.225 −0.065 −0.140 0.030 −0.063 **−0.473** −0.164 0.011
*KSU*	B1(A1) 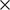 C1 versus B1(A1) 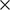 C2 B1(A1) 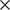 C1 versus B2(A1) 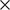 C2 B1(A1) 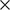 C1 versus B3(A2) 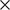 C2* B1(A1) 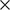 C1 versus B4(A2) 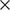 C2* B2(A1) 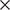 C1 versus B1(A1) 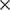 C2 **B2(A1)** 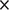 **C1 versus B2(A1)** 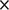 **C2** B2(A1) 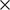 C1 versus B3(A2) 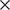 C2* B2(A1) 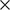 C1 versus B4(A2) 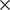 C2*	−2.038 −0.741 0.879 0.373 −0.436 **−6.976** 0.746 0.224	0.461 0.996 0.987 ∼1 ∼1 **<0.001** 0.995 ∼1	∼1 ∼1 ∼1 ∼1 ∼1 **<0.001** ∼1 ∼1	−0.173 −0.063 0.075 0.032 −0.037 **−0.594** −0.064 0.019

*Note:* *simple interaction multilevel effects; **t-test was corrected for multiple comparisons. Important warning: All means comparisons come from matrix annotations of Table 3.

Abbreviations: DV = dependent variables; BA = BAI affective anxiety; BS = BAI somatic anxiety; KS = S-KUAS somatic anxiety; KC = S-KUAS cognitive anxiety; KSU = S-KUAS subjective anxiety; d = Cohen’s d corrected using Hedges’ g.

**Table 9.** Simple Main and Interaction Effects Analysis for the Nonbelievers

DV	Means comparison	t-test**	*p* values (Tukey)	*p* values (Bonferroni)	d
*BA*	B3(A2) 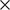 C1 versus B1(A1) 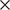 C2* B3(A2) 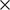 C1 versus B2(A1) 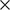 C2* B3(A2) 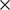 C1 versus B3(A2) 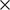 C2 B3(A2) 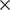 C1 versus B4(A2) 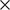 C2 B4(A2) 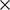 C1 versus B1(A1) 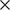 C2* B4(A2) 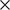 C1 versus B2(A1) 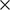 C2* B4(A2) 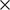 C1 versus B3(A2) 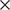 C2 B4(A2) 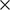 C1 versus B4(A2) 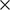 C2	−0.082 −0.649 −0.369 0.706 −0.907 −1.507 −0.907 −0.537	∼1 0.993 ∼1 0.997 0.985 0.803 0.985 0.999	∼1 ∼1 ∼1 ∼1 ∼1 ∼1 ∼1 ∼1	−0.007 −0.055 −0.031 0.060 −0.077 −0.128 −0.077 −0.046
*BS*	B3(A2) 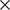 C1 versus B1(A1) 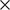 C2* B3(A2) 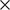 C1 versus B2(A1) 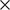 C2* B3(A2) 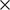 C1 versus B3(A2) 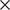 C2 B3(A2) 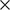 C1 versus B4(A2) 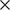 C2 B4(A2) 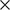 C1 versus B1(A1) 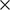 C2* B4(A2) 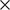 C1 versus B2(A1) 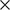 C2* B4(A2) 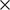 C1 versus B3(A2) 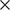 C2 B4(A2) 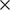 C1 versus B4(A2) 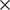 C2	0.250 0.032 −2.208 −1.238 1.133 0.938 0.520 −2.270	∼1 ∼1 0.354 0.919 0.948 0.982 ∼1 0.318	∼1 ∼1 0.811 ∼1 ∼1 ∼1 ∼1 0.685	0.021 0.003 −0.188 −0.105 0.096 0.080 0.044 −0.193

*Note:* *simple interaction multilevel effects; **t-test was corrected for multiple comparisons. Important warning: All means comparisons come from matrix annotations of Table 3.

Abbreviations: DV = dependent variables; BA = BAI affective anxiety; BS = BAI somatic anxiety; KS = S-KUAS somatic anxiety; KC = S-KUAS cognitive anxiety; KSU = S-KUAS subjective anxiety; d = Cohen’s d corrected using Hedges’ g.

**Table 10.** Simple Main and Interaction Effects Analysis for the Nonbelievers (continuation Table 9)

DV	Means comparison	t-test**	*p* values (Tukey)	*p* values (Bonferroni)	d
*KS*	B3(A2) 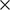 C1 versus B1(A1) 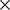 C2* B3(A2) 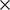 C1 versus B2(A1) 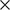 C2* B3(A2) 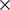 C1 versus B3(A2) 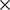 C2 B3(A2) 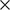 C1 versus B4(A2) 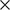 C2 B4(A2) 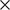 C1 versus B1(A1) 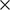 C2* B4(A2) 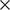 C1 versus B2(A1) 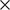 C2* B4(A2) 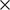 C1 versus B3(A2) 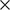 C2 B4(A2) 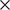 C1 versus B4(A2) 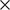 C2	−0.963 −1.001 −1.047 −0.451 −1.235 −1.282 −0.559 −2.541	0.973 0.974 0.962 ∼1 0.920 0.904 0.999 0.188	∼1 ∼1 ∼1 ∼1 ∼1 ∼1 ∼1 0.341	−0.082 −0.085 −0.089 −0.038 −0.105 −0.103 −0.048 −0.216
*KC*	B3(A2) 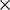 C1 versus B1(A1) 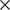 C2* B3(A2) 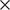 C1 versus B2(A1) 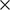 C2* B3(A2) 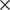 C1 versus B3(A2) 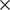 C2 B3(A2) 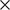 C1 versus B4(A2) 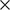 C2 B4(A2) 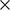 C1 versus B1(A1) 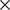 C2* B4(A2) 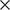 C1 versus B2(A1) 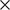 C2* B4(A2) 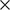 C1 versus B3(A2) 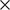 C2 B4(A2) 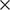 C1 versus B4(A2) 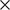 C2	0.936 0.702 −1.218 1.799 −0.992 −1.287 −2.171 −0.788	0.982 0.997 0.925 0.622 0.975 0.902 0.376 0.993	∼1 ∼1 ∼1 ∼1 ∼1 ∼1 0.886 ∼1	0.080 0.062 −0.104 0.153 −0.084 −0.110 −0.185 −0.067
*KSU*	B3(A2) 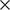 C1 versus B1(A1) 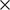 C2* B3(A2) 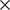 C1 versus B2(A1) 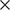 C2* B3(A2) 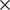 C1 versus B3(A2) 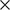 C2 B3(A2) 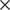 C1 versus B4(A2) 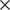 C2 B4(A2) 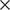 C1 versus B1(A1) 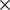 C2* B4(A2) 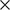 C1 versus B2(A1) 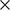 C2* B4(A2) 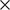 C1 versus B3(A2) 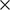 C2 B4(A2) 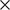 C1 versus B4(A2) 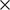 C2	−1.285 −1.784 −1.019 −0.656 −0.745 −1.237 0.421 −0.742	0.903 0.632 0.971 0.998 0.995 0.919 ∼1 0.996	∼1 ∼1 ∼1 ∼1 ∼1 ∼1 ∼1 ∼1	−0.109 −0.152 −0.087 −0.056 −0.063 −0.105 0.036 0.063

*Note:* *simple interaction multilevel effects; **t test was corrected for multiple comparisons. Important warning: All means comparisons come from matrix annotations of Table 3.

Abbreviations: DV = dependent variables; BA = BAI affective anxiety; BS = BAI somatic anxiety; KS = S-KUAS somatic anxiety; KC = S-KUAS cognitive anxiety; KSU = S-KUAS subjective anxiety; d = Cohen’s d corrected using Hedges’ g.

No significant results were obtained in the analyses of simple effects for the comparisons of “nonbelievers.” If the healing crystals had observable therapeutic effects in reducing anxiety, the differences between the pre- and post-means of the experimental participants nested in the category of nonbelievers should also have been significant. Similarly, the effects of multilevel interaction (these effects are marked with an asterisk in Tables 7 through 10) were also not significant in any dependent variable. For example, the differences were not significant when the pre-measures of the nested control participants in the “believers” category were compared with the post scores of the nested control participants in the “nonbelievers” category. **This indicated that, if there were a placebo effect, it would not be a truly significant effect.** The same reasoning applies to pre-experimental participants nested in the category “believers” compared to post-experimental participants nested in the category “nonbelievers.” **If healing crystals had healing properties, significant differences should be observed in these comparisons of simple multilevel effects.**

**Figure 6.**
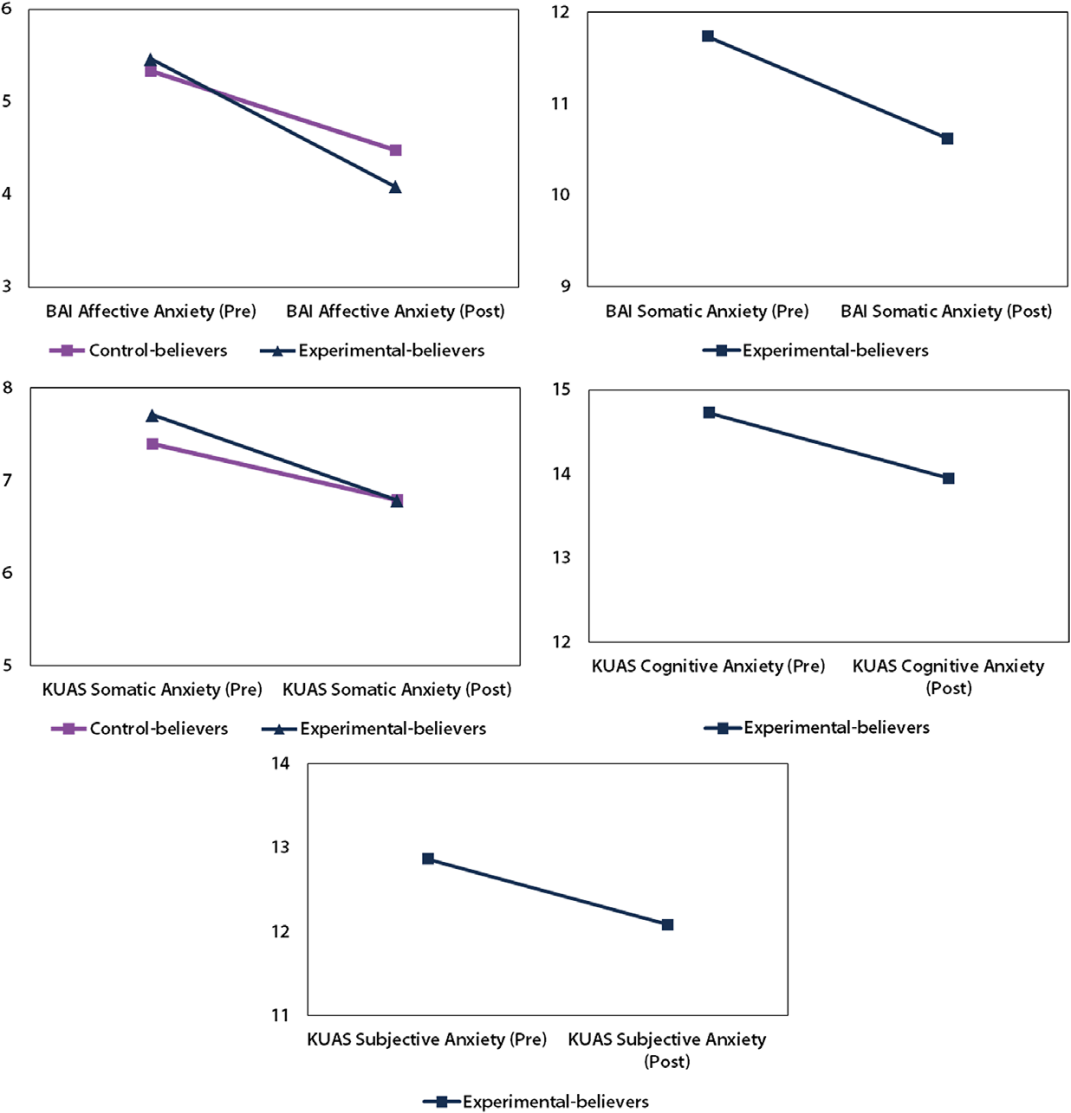
Graph of means of anxiety levels comparing the groups of the experiment. Each graph specifies whether anxiety levels were measured with the BAI or the KUAS.

The only significant differences observed indicated that means tend to decrease after the use of healing crystals. These trends are illustrated in Figure 6. The interpretation of which theory or behavioral model explains these declines is developed in the discussion.

### Analysis of classical conditioning and causal illusions

To check whether the associations described in Figure 2 were fulfilled in the study participants, the answers to the pre- and post-“treatment” questions regarding beliefs about whether the healing crystals would work were correlated. Table 11 provides the statistics relating to the correlation coefficients and regression.

**Table 11.** Correlation and Regression Model between Pre- and Post-Concerning Healing Crystals Effectiveness Beliefs

	Previous beliefs		Beliefs after experience		*Fisher’s F* (*df_1_; df_2_*)	r	β	R^2^
	*M*	*SD*	*M*	*SD*				
A1	7.93	1.255	6.4	1.527	100.127** (1; 68)	0.772**	0.939 (−1.044)	0.59
A2	2.47	0.969	1.63	0.913	16.908** (1; 66)	0.452**	0.425 (0.582)	0.192
Total	5.24	2.958	4.05	2.703	977.604** (1; 136)	0.937**	0.854 (−0.433)	0.877

*Note:* M = means; SD = standard deviation; **p < 0.001; r = Pearson’s correlation coefficient; A1 = believers group; A2 = nonbelievers group; β = regression coefficient (intercept is in brackets); R^2^ = determination coefficient adjusted or explained variance.

A total positive linear relationship was obtained. Pre-test responses predicted 87.7% post-test responses. This means that the participants applied the classical conditioning models through the *prophecy of self-fulfillment*; as they developed higher expectations about how the healing crystals worked, they became more convinced that they had “worked.”

## Discussion

This study evaluated the supposed therapeutic effects of healing crystals using explanations based on classical conditioning theories. Multilevel contrasts indicated that healing crystals only have therapeutic effects for believing participants. These effects did not exceed the placebo effect estimated for the CGs. Given that differences were only observed at level A1 (corresponding to the category “believers”), it was concluded that the model of classical conditioning illustrated in Figures 1 and 2 satisfactorily explained the change in score trends.

### Interpretation of results according to the theory of classical conditioning

The application of classical conditioning as an explanation derived from *cognitive dual process theory.* ^39^ If healing crystals really had healing properties, the simple effects of multilevel interaction would have been significant. This is true, except for the placebo effect, since scientific objectivism prevents the acceptance of such effects being significant only for those who believe in the power of healing crystals.

The main mechanism of classical conditioning that justifies why the results described above were obtained lies in the conditioned stimulus or cognition, “the crystal works”.^4^ This conditioned stimulus is associated with the neutral stimulus “rose quartz,” and the successive expositions, repetitions, and associations between NS + CS trigger a conditioned response inhibiting the unconditioned anxiety levels recorded as “baseline.”

It should be noted that the crystal works stimulus is a conditioned stimulus because it represents a cognitive attribution resulting from the intuitive processing of information and the magical beliefs assumed by the subject.^40^ Therefore, following dual processing theory,^39^ if the subject does not develop intuitive cognitive styles, it is difficult for them to have magical beliefs that enable the establishment of the association NS (“rose quartz”) + CS (“the crystal works”) = UR (“relaxation”). In fact, this association should justify the placebo effect observed in the results.

According to the model of classical second-order conditioning, the association between NS + CS = UR required the causal attribution analyzed in section “***Analysis of classical conditioning and causal illusions***”. of the results. The answers to the questions on expectations show that the causal illusions are developed by applying the prophecy of self-fulfillment^49^: The more a subject believes that the rose quartz works, the greater the causal illusion and the more they are convinced that the healing crystals work. This coincides with the theories proposed by Matute,^40^ ^,^^50^ in which causal illusions explain why some participants believe that pseudoscientific therapies “work.” However, the regression applied to both groups (believers and nonbelievers) indicated that causal illusions are a bias that affects both types of thinking (intuitive and critical-analytical), although believers experience this bias more often.

These findings signify that classical condition is an essential procedure in the development of causal illusions and vice versa.^51^ Causal illusions are also explained by classical and instrumental conditioning^36^ ^,^^52^ because they derive from learning theory principles. Specifically, the model of classical conditioning would appear when there was an erroneous association, such as NS + US/CS that induces an erroneous causal attribution. This could be understood as “bad learning” and the counter-conditioning models should be applied.^4^ ^,^^5^

### Not just illusions: the benefits of the placebo effect

While classical learning theory through conditioning may help explain part of the placebo effects observed in alternative interventions like the one used in this study, the mere fact that these effects can be interpreted as a form of illusory learning does not render them false or devoid of value. To conclude that the placebo effect we identified is solely the result of causal illusions would be reductive, for two key reasons: (1) beliefs regarding the efficacy of rose quartz – whether favorable or skeptical – were not variables directly manipulated by the researchers. Instead, they were based on participants’ self-reported beliefs, which determined group assignment. Randomization was applied to the type of intervention, not to belief levels, which calls for interpretive caution, and (2) even if illusion-related phenomena may appear clinically unpromising, in practice, they can occasionally serve useful functions. Some studies have reported meaningful benefits associated with placebo effects that should not be dismissed.^53^

For example, in a broader context, when conventional psychiatric treatments have failed and patients lack access to alternative evidence-based care, clinicians have sometimes employed alternative interventions primarily for their placebo effect. This decision often stems not from the clinician’s belief in the intervention itself, but from its potential to generate subjective well-being for the patient.^54^ Provided that there is no harm involved, these practices suggest that placebo effects may offer a legitimate avenue for clinical benefit. In line with this, recent medical research has argued for a more open attitude toward using placebo effects as minimally effective clinical tools, particularly for the psychological relief they may provide.^55^ For instance, certain standard psychological treatments for stress during the COVID-19 crisis were found to be no more effective than placebo effects conditioned by patient expectations prior to treatment – yet these interventions still yielded measurable benefits.^56^

In clinical psychology, one widely used strategy to minimize expectancy-driven effects is the establishment of a strong therapeutic alliance between clinician and patient.^57^ There is evidence indicating that the therapeutic alliance and placebo effect can interact, with the doctor–patient relationship reinforcing placebo-induced improvements.^58^ This brings us to a simple yet challenging question: Although rose quartz lacks any classically detectable therapeutic effect beyond placebo, could that placebo effect be modulated by an effective therapeutic alliance? This is a question that has received little consideration in clinical mental health care. Alternative techniques used outside a structured therapeutic context – one grounded in the clinician–patient bond – may amount to little more than illusions or inert placebo. But it is equally important to consider whether this same bond could enhance the placebo response, transforming an otherwise negligible effect into a potentially therapeutic one.

We propose, consistent with findings from other placebo-related studies in mental health,^59^ that placebo effects alone may offer limited utility. However, when they interact with contextual and affective variables that define the doctor–patient relationship, their impact may be significantly amplified. Future research should explore how the mechanisms underlying causal illusions might be harnessed for therapeutic benefit – shifting their role from clinical risk to clinical resource.

As researchers, we do not take a position for or against alternative therapies. Rather, we are willing to entertain unconventional and divergent hypotheses like the one presented here. If this subsection causes confusion for certain readers or professionals, it is important to clarify: We do not offer this speculation as an endorsement of alternative therapies, but as a scientifically testable hypothesis that logically extends from the present study – however unlikely it may initially appear. Furthermore, as previously cited,^55^ there are empirical studies pointing in this direction, offering not only theoretical reasoning but also data that support our proposal.

### Criticisms and limitations

Three main limitations can be highlighted in this research:

(1) The sample used came from the general population. It would have been ideal to work with clinical participants who were diagnosed with distress and anxiety symptoms. This criticism raises the following caveat: The effect size values of single effects may be small because the symptoms of anxiety that were assessed were not clinically elevated, and therefore, a broad reduction in symptoms should not be expected.

(2) The application of classical conditioning was not carried out through experimental manipulation. This means that the CS (ie, “the crystal works”) could not be experimentally controlled during the course of the study. As such, this remains an inference or a hypothetical assumption rather than direct evidence. Nonetheless, the distinction between believers and nonbelievers, along with the results obtained, provides reasonable support for this possibility.

In relation to this limitation, it is important to acknowledge that belief systems are shaped by individual and cultural differences. We raise this point because future studies aiming to build on this line of research may benefit from including statistical controls to assess the extent to which personal and sociocultural characteristics influence variation or changes in the placebo effect. Such an approach could take us a step further in understanding how the act of believing may acquire different qualities depending on context, and how this, in turn, may affect placebo responses. Ideally, direct experimental manipulation of belief acquisition would offer the most robust means of controlling for these effects. However, a major challenge lies in the fact that no adult is entirely decontextualized – everyone brings prior experiences that establish a baseline, making it difficult to achieve statistical equivalence across participants. Therefore, we recommend the use of cross-cultural comparisons and statistical controls wherever possible, particularly when working with samples drawn from diverse populations.

Finally (3), for the placebo effect, glass stones were used that accurately simulated rose quartz. The problem is that the participants could consult an expert or know by themselves that it was not rose quartz, but an imitation, although the imitation was of quality. This does not represent a methodological error, since the instructions explicitly stated, “do not allow other people to see or touch it,” and participants who did not comply with the instructions should indicate this to the researcher. Therefore, more than an error, this is a limitation because it is an “act of faith” that the researcher had to perform.

Considering these limitations, in future research lines, the supposed effects of pseudoscientific therapies should be analyzed and replicated using a clinical sample, exercising experimental control of the classical conditioning model used, and ensuring a placebo-substance that prevents the participant’s checking of the crystal’s authenticity.

### Conclusions

The results and analysis applied in this research lead to the following conclusions: (1) In the contrasts made, no significant effects were observed that could support the supposed “efficacy” of healing crystals in the anxiety level reductions over the placebo effect. (2) The significant reductions observed in anxiety levels are equivalent to the reductions observed for the estimation of the placebo effect applied in the CG. (3) The classical conditioning theory can explain the differences that were found, since such significant results were only obtained for the pre and post comparisons in the control and experimental participants nested in the category “believers.” (4) Belief systems play an essential role in the placebo effect generation, since it is an effect related to the prophecy of self-fulfilling belief. (5) Causal illusions can be explained by classical conditioning. Classical conditioning represents the theoretical scientific basis for the causal illusions and the placebo effect in this context. Finally (6), the fact that classical conditioning can be applied as the main explanation for the supposed efficacy of pseudoscientific therapies does not detract from the possible usefulness of the placebo effect of healing crystals.

This final conclusion is perhaps the most significant of all. Based on previously published evidence,^55^ if the placebo effect interacts with other clinical variables – such as the therapeutic alliance between doctor and patient – it could be amplified, becoming a powerful tool for addressing certain forms of distress and suffering, including stress. Integrating the therapeutic alliance with potential placebo effects opens a promising new avenue for research into alternative therapies. Even if such therapies can be explained by classical conditioning or causal illusions, these mechanisms would serve the goal of enhancing well-being. In this light, they shift from being potential risk factors to becoming meaningful components of the healing process – part of the treatment, not the illness.

## Raw data access statement

The data will be available upon request to the author of the manuscript, provided that properly justified reasons are given.
